# Nomenclature in laboratory robotics and automation (IUPAC Recommendation 1994)

**DOI:** 10.1155/S1463924694000040

**Published:** 1994

**Authors:** H. M. (Skip) Kingston, M. L. Kingstonz

**Affiliations:** 1 Department of Chemistry Duquesne University Pittsburgh PA 15282 USA; 2 OCLC 212 Elmhurst Circle Cranberry Township PA 16066 USA

## Abstract

These recommended terms have been prepared to help provide a uniform approach to terminology and notation in laboratory automation and robotics. Since the terminology used in laboratory automation and robotics has been derived from diverse backgrounds, it is often vague, imprecise, and in some cases, in conflict with classical automation and robotic nomenclature.

These dejinitions have been assembled from standards, monographs, dictionaries, journal articles, and documents of international organizations emphasizing laboratory and industrial automation and robotics. When appropriate, definitions have been taken directly from the original source and identified with that source. However, in some cases no acceptable definition could be found and a new definition was prepared to define the object, term, or action. Attention has been given to defining specific robot types, coordinate systems, parameters, attributes, communication protocols and associated workstations and hardware. Diagrams are included to illustrate specific concepts that can best be understood by visualization.


Journal of Automatic Chemistry, Vol. 16, No. 2 (March–April 1994), pp. 43–57

*International Union of Pure and Applied Chemistry*
*Analytical Chemistry Division*
*Commission on Analytical Nomenclature**
*Commission on General Aspects of Analytical Chemistry*†

### Nomenclature in laboratory robotics and automation

**(IUPAC Recommendations 1994)**
**Prepared for publication by**
**H. M. (Skip) Kingston[1] and M. L. Kingston[2]**
[1] *Department of Chemistry, Duquesne University, Pittsburgh, PA 15282, USA*
[2] *OCLC, 212 Elmhurst Circle, Cranberry Township, PA 16066, USA*

* Membership of the Commission during the period (1987–1989) when this report was being prepared is given hereunder (*Note: The Commission ceased to exist after the 35th IUPAC General Assembly, Lund, 1989.*)

*Chairman:* R. E. Van Grieken (Belgium); *Secretary:* C. L. Graham (UK); *Titular members:* C. A. M. G. Cramers (Netherlands), L. A. Currie (USA), W. Horwitz (USA), D. Klockow (FRG), M. Parkany (Switzerland); Associate Members: L. S. Ettre (USA), D. M. Everaerts (Netherlands), Y. Gohshi (Japan), P. S. Goel (India), H. M. Kingston (USA), G. J. Patriarche (Belgium), D. L. Rabenstein (USA), J. W. Stahl (USA); National Representatives: Magda Ariel (Israel), K. Danzer (GDR), U. L. Haldna (USSR), D. R. Reeves (New Zealand), B. Schreiber (Switzerland), J. Stary (Czechoslovakia), G. Svehla (UK).

† *Membership of the Commission during 1989–91 was as follows:*

*Chairman:* F. Ingman (Sweden); *Vice-chairman:* R. E. Van Grieken (Belgium); *Secretary:* W. E. van der Linden (Netherlands); Co-Secretary: C. L. Graham (UK); *Titular members:* L. A. Currie (USA); St. Glab (Poland); W. Horwitz (USA); D. L. Massart (Belgium); M. Parkany (Switzerland) Associate Members: P. S. Goel (India); Y. Gohshi (Japan); H. Muller (FRG); M. Otto (FRG); G. J. Patriarche (Belgium); S. B. Savvin (USSR); J. W. Stahl (USA); P. J. Worsfold (UK); National Representatives: Tania M. Tavares (Brazil); L. Sommer (Czechoslovakia); D. Klochow (FRG); K. Danzer (GDR); J. Inczédy (Hungary); D. Thorburn Burns (Ireland); R. D. Reeves (New Zealand); A. Hulanicki (Poland); B. Schreiber (Switzerland); S. Ates (Turkey); G. Svehla (UK).

*Membership of the Commission during 1991–93 was as follows:*

*Chairman:* W. E. van der Linden (Netherlands); *Secretary:* C. L. Graham (UK); *Titular members:* L. A. Currie (USA); St. Glab (Poland); W. Horwitz (USA); D. L. Massart (Belgium); M. Parkany (Switzerland) Associate Members: K. Danzer (GDR); Y. Gohshi (Japan); H. Muller (FRG); M. Otto (FRG); J. W. Stahl (USA); P. J. Worsfold (UK); National Representatives: Tania M. Tavares (Brazil); J. Garaj (Czechoslovakia); J. Inczédy (Hungary); D. Thorburn Burns (Ireland); R. D. Reeves (New Zealand); J. F. van Staden (RSA); F. Ingman (Sweden); S. Ates (Turkey).

Names of countries given after members' names are in accordance with the *IUPAC Handbook 1991*–93; changes will be effected in the 1994–95 edition.

*Republication of this report is permitted without the need for formal IUPAC permission on condition that an acknowledgment, with full reference together with IUPAC copyright symbol (© 1994 IUPAC), is printed. Publication of a translation into another language is subject to the additional condition of prior approval from the relevant IUPAC National Adhering Organization.*

### Synopsis

*These recommended terms have been prepared to help provide a uniform approach to terminology and notation in laboratory automation and robotics. Since the terminology used in laboratory automation and robotics has been derived from diverse backgrounds, it is often vague, imprecise, and in some cases, in conflict with classical automation and robotic nomenclature.*

*These definitions have been assembled from standards, monographs, dictionaries, journal articles, and documents of international organizations emphasizing laboratory and industrial automation and robotics. When appropriate, definitions have been taken directly from the original source and identified with that source. However, in some cases no acceptable definition could be found and a new definition was prepared to define the object, term, or action. Attention has been given to defining specific robot types, coordinate systems, parameters, attributes, communication protocols and associated workstations and hardware. Diagrams are included to illustrate specific concepts that can best be understood by visualization.*

© 1994 IUPAC

**These assembled definitions will be useful for the practitioners of laboratory automation and robotics.**

### Introduction

Commercial laboratory robots were first introduced at the 1982 Pittsburgh Conference, although pick-and-place robots had been used previously. Although laboratory automation efforts have been developing in various ways for many years, it was the introduction and implementation of robotics to the chemical laboratory that focused the efort and provided a reference point.

Laboratory robotics is not an outgrowth of classical industrial robotics (manufacturing robotics). Developed independently, it is oriented more toward the chemical process rather than centred around robotic hardware development. However, much of the technology that has been developed and tested for industrial automation is finding application in laboratory robotics. In some cases, classical terms have been applied to laboratory robotics and laboratory automation efforts. Terms have also developed to describe devices and processes unique to laboratory automation. Therefore, laboratory robotics and automation has unique terms, as well as terms shared with classical robotics and automation.

As with most scientific endeavours, many standards are relied upon to accomplish instrumental harmony of the laboratory automation system. Many of these terms, standards, and protocols are provided as a reference for chemical laboratory automation efforts.

This assembly of terms was compiled from recent laboratory automation and robotic references and classical compilations.

### Glossary

*Absolute accuracy (in robotics)*
The difference in position between a point called for by a robot's control system and the point actually achieved by the robot. The tolerance in each co-ordinate in reaching anv given point in space [2].

*Absolute location*
A location in the robot's work envelope defined by specific coordinates. See also *Relative location.*

*Acceleration*
Rate of change of the velocity at the point under consideration per unit of time [21].

*Accuracy (in robotics)*
The degree to which actual position corresponds to desired or commanded position; the degree of freedom from error. Accuracy involves the capability to hit the mark, or reach the point in space, or get the correct answer; repeatability is the ability to duplicate an action or a result every time. Accuracy of a robot is achieved (or lost) by three elements of the system: the resolution of the control system, the inaccuracies or imprecesion of the mechanical linkages and gears and beam deflections under different load conditions, and the minimum error that must be tolerated to operate the arm under closed servoloop operation. Accuracy refers to the degree of closeness to a 'correct' value; precision refers to the degree of preciseness of a measurement. Frequently confused with precision [20].

*Active accommodation*
Integration of sensors, control, and robot motion to achieve alteration of a robot's preprogrammed motions in response to sensed forces. Used to stop a robot when forces reach set levels, or to perform force feedback tasks like insertions, door opening and edge tracing [5].

*Actuator*
(1) A power mechanism used to effect motion of the robot [39].
(2) A motor or transducer that converts electrical, hydraulic, or pneumatic energy into motion.

*Adaptive control*
A control algorithm or technique in which the controller can change its control parameters and performance characteristics in response to its environment and experience. (Modified from reference [5].)

*Algorithm*
A prescribed set of well-defined rules, processes, mathematical equations, or programmed sequence of instructions for the solution of a problem in a finite number of steps.

*Analogue*
The representation of a smoothly changing physical variable by another physical variable. In data transmission, the term is used in contrast to digital. In this context, analogue transmission uses amplifiers, required due to attenuation of the signal with distance, that magnify the incoming signal. (Modified from reference [20].)

*Analogue data*
Data represented by a physical quantity that is considered to be continuously variable and whose magnitude is made directly proportional to the data or to a suitable function of the data [32].

*Analytical automation module*, see *Module*

*Analytical instrument*
A device or combination of devices used to carry out an analytical process [29].

*Archive*
The storage of information for future use [1].

*Arm*
An interconnected set of links and powered joints comprising a manipulator that supports or moves a wrist or end-effector [4].

*Articulated structure*
Set of links and joints that constitute the arm and the wrist [39].

*Articulation*
The manner and actions of jointing in a robot. The greater the number, the easier it is for a robot to move and attain any position. Types of articulations are fixed beam, linear joint, ball joint, round joint, revolute or pin joint, and others. They vary in the number of degrees of freedom [2].

*Artificial intelligence*
The capability of a machine to perform human-like intelligence functions, such as learning, adapting, reasoning and self-correction. The main areas of application are currently in expert systems, computer vision, natural language processing, robotics, and speed synthesis and recognition.

*ASCII*
Abbreviation for American Standard Code for Information Interchange. It is an eight-bit (seven bits plus an optional parity bit) code for representing alphanumerics, punctuation, and certain special characters for control purposes. (Modified from reference [5].)

*Automate*
To replace human manipulative effort and faculties in the performance of a given process by mechanical and instrumental devices which are regulated by feedback of information, so that the apparatus is self-monitoring or self-adjusting [36].

*Automated analytical system*
A collection of analytical automation modules and modular analytical instruments configured to automate a complete analysis, from sample input to information output. The analytical system contains a user-interface to permit human interaction and may also have an archival module to provide the audit trail. (Modified from reference [28].)

*Automatic end-effector exchanger*
A coupling device between the mechanical interface of the robot and the end-effector enabling automatic exchange of end-effectors [39].

*Automation*
The use of combinations of mechanical and instrumental devices to replace, refine, extend, or supplement human effort and faculties in the performance of a given process, in which at least one major operation is controlled, without human intervention, by a feedback system [36].

*Axis*
A direction used to specify the robot motion in a linear or rotary mode [39].

*Balance interface*
The electronic hardware and control software necessary to utilize an electronic balance as an automated workstation. (Modified from reference [1].)

*Bar code*
An identification symbol in which the symbol value is encoded in a sequence of high-contrast bars and spaces. The relative widths of the bars and spaces contain the information. This machine-readable code, often on a label, is read (usually optically by a light pen or a laser scanner) into a decoder which transmits the encoded information to some external device for display, storage, or conditional processing.

*Base (in robotics)*
The platform or structure to which is attached the origin of the first member of the articulated structure [21].

*Base co-ordinate system*
A co-ordinate system referenced to the base of the robot [21].

*Base mounting surface*
The connection surface between the robot and its periphery upon which is defined the base co-ordinate system [21].

*Batch processing*
A technique in which a number of similar data or transactions are collected over a period of time and aggregated (batched) for sequential processing as a group during a machine run [20].

*Baud rate*
The measure of speed of signal transmission in data communications. The term 'baud' refers to the number of times the line condition changes state per second. It can be measured in signal events (bits) per second. (Modified from reference [20].)

*Bit*
Either of the digits 0 or 1 when used in the pure binary numeration system [42].

*Branching*
The function of a computer program that alters the logic path, depending on some detected condition or data status. For example, the program would branch to a reorder routine when the projected available went negative [20].

*Bug*
A mistake, omission or error in a computer program usually leading to unexpected or undesired actions or occurrences.

*Bus*
(1) A facility for transferring data between several devices located between two end points, only one device being able to transmit at a given moment [34].
(2) Conductor or group of conductors used to trasmit signals or power. An information-coding method for signals on a common data channel. (Modified from reference [2].)

*Byte*
A string that consists of eight bits [42].

*CAALS-I communication specification*
A standard specification for message interchange between modules and a controller that specifies the communication protocol and message syntax, but not the semantics of the messages. (Modified from reference [46].)

*Calibration*
(1) The correction of the deviation from a standard [41].
(2) To determine the deviation from a standard so as to ascertain the proper corrections [41].

*Capability dataset*
A collection of information describing the characteristics, capabilities, and idiosyncratic behaviors of a module. This information may be static (for example default settings, software revision, device type, etc.) or dynamic (for example date of last calibration, number of samples currently in queue, etc.). (Modified from reference [45].)

*Capping station*
A laboratory workstation that is used to cap and uncap sample containers with a cap [1].

*Cartesian co-ordinate robot*
A robot whose joints travel in right angle lines to each other. There are no radial motions. The profile of its work envelope represents a rectangular shape. Also referred to as gantry robot. (Modified from reference [5].)

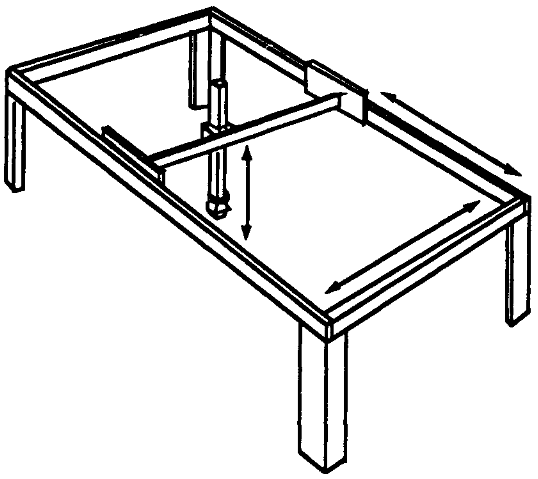

*Figure 1. Cartesian or rectangular robot – gantry robot.*

*Cartesian co-ordinate system*
A co-ordinate system with axes or dimensions that are intersecting and perpendicular (orthogonal). The origin is the intersection of the three co-ordinates-*x*, *y* and *z* axes-that locate a point in space and measure its distance from any of three intersecting co-ordinate planes. The coordinates are used to identify points for the positioning of an end-effector [2].

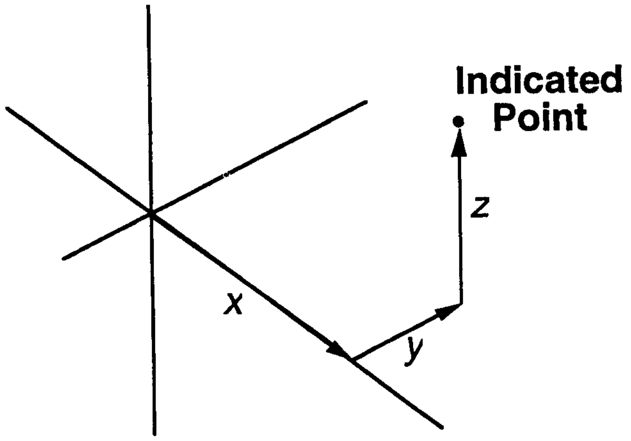

*Figure 2. Cartesian co-ordinate system.*

*Chemical analysis system*
A combination of laboratory modules that complete sample preparation, analysis, and evaluation to provide chemical information. (Modified from reference [28].)

*Closed-loop control*
Control achieved by feedback, i.e., by measuring the degree to which actual system response conforms to desired system response and utilizing the difference to drive the system into conformance. The measurement of the difference between what is achieved and what is asked for is used to increase accuracy and reliability.

*Command*
An instruction to the robot's controller given in a language or form that the controller can understand. (Modified from reference [1].)

*Command pose*
The pose specified by the task program.

*Compliance*
The flexible behavior of a robot or any associated tool in response to external forces exerted on it [39].

*Computer interface*
An interconnection which allows an electronic device to send data to or receive data from a computer.

*Computer network*
A network of data processing nodes that are interconnected for the purpose of data communication [35].

*Concurrent processing*
The simultaneous processing of more than one program [20].

*Configuration*
(1) A set of joint displacements values, equal in number to the number of primary axes, that completely determine the shape of the arm at any time [39].
(2) The arrangement of control and peripheral devices at a robotic site. The type of manipulator being used in an application: Cartesian co-ordinate, cylindrical co-ordinate, jointed-arm, or spherical co-ordinate [2].

*Confirmation technique*
A technique used in automation to verify that some event has or has not taken place. This usually incorporates taking a preplanned corrective action if a problem has been sensed. This technique may depend on the use of contact sensors, proximity sensors, external sensors, or other sensors.

*Contact sensor*
A device capable of sensing mechanical contact [5].

*Continuous path control*
A control procedure whereby the user can impose to the robot the path to be followed between commanded poses at a programmed velocity [39].

*Controlled-path robot*
A robot whose path, contour, and/or speed are programmed. End points are programmed and the computer automatically creates the robot's path. This robot is taught its motions according to capabilities inherent in point-to-point and continuous-path systems: robot axes need not be specified, while the desired contour, acceleration, and deceleration are automatically generated. Special features of this kind of robot are path computations, programmable velocities, coordinated axis motions, ability to make changes in end-effector length, use of multi-robots, mirror imaging, and software editing and diagnosis. (Modified from reference [2].)

*Controller*
An information processing device whose inputs are both desired and measured position, velocity or other pertinent variables in a process and whose outputs are drive signals to a controlling motor or actuator [41].

*Co-ordinated axis control*
Control in which the axes of a robot arrive at their end points at the same time, thus giving a smooth appearance to any motion. Control in which the motions of the axes are such that the end point moves along a specific type of path or contour [2].

*Corrosion resistant robot*
A robot that has been modified for use in mildly corrosive environments. Modification includes replacement of exposed, corrosion susceptible parts with more corrosion resistant materials and gas lines for purging the robot electronics enclosures (Modified from reference [1].)

*Crash*
A breakdown resulting from software or hardware malfunction [20].

*Crimp capping station*
An automated workstation used to crimp retaining caps onto glass vials such as those used in chromatographic analysis.

*Cybernetics*
The study of control and communication in, and particularly between, humans and machines. A human-machine cybernetic system is a functional synthesis of a human system and a technological system or machine.

*Cycle*
A single execution of a complete set of moves and functions contained within a robot's program [41].

*Cycle time*

Time required to perform the cycle [39].

*Cylindrical robot*
A robot whose arm has at least one rotary and at least one prismatic joint and whose axes form a cylindrical co-ordinate system [39].

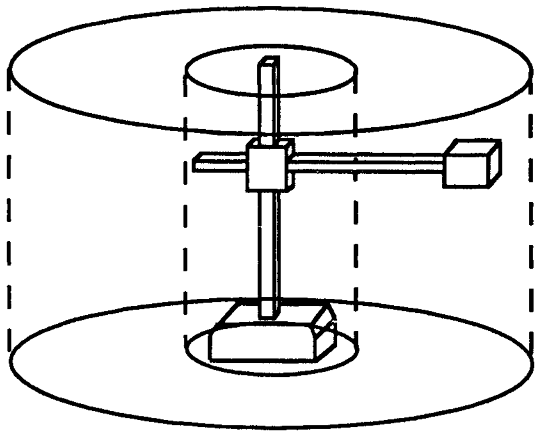

*Figure 3. Cylindrical robot and cylindrical work envelope.*

*Cylindrical co-ordinate system*
A co-ordinate system that defines the position of any point in terms of an angular dimension, a radial dimension, and a height from a reference plane. These three dimensions specify a point in a cylinder [5].

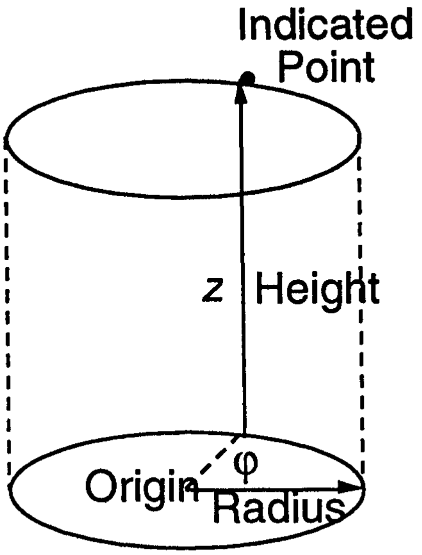

*Figure 4. Cylindrical co-ordinate system.*

*Database*
A comprehensive collection of interrelated information stored on some form of mass data storage device. Generally consists of information organized into a number of fixed-format record types with logical links between associated records. (Modified from reference [20].)

*Decomposition*
In control hierarchy, the breakdown of higher-level tasks into sets of procedurally simpler ones. These simpler tasks, in turn, become the goals of other tasks in a lower level of the control system. In the architecture of a control system that is hierarchically arranged, each level of the control receives inputs and produces outputs that, in their turn, become inputs to another level of control [2].

*Degree of freedom*
One of the variables (maximum number of six) required to define the motion of a body in space [39].

*Digital control*
Control involving digital logic devices that may or may not be complete digital computers [5].

*Digital data*
Data represented by digits, perhaps with special characters and the space character [32].

*Distal*
Away from the base, toward the end-effector of the arm [5].

*DOF* see *Degree of freedom*

*Drift (in robotics)*
The tendency of a system's response to gradually move away from the desired response with time. (Modified from reference [5].)

*Drive system*
The source of the robot's locomotion, such as stepping motors, servomotors, pneumatic or hydraulic power. (Modified from reference [1].)

*Dual function hand*
An end-effector that combines the functions of multiple hands or end-effectors, such as a general purpose gripper and a liquid dispensing hand.

*Duty cycle*
The fraction of time during which a device or system will be active, or at full power [5].

*Dynamic*
A state in which an entity changes with time [41].

*Echo check*
A method of checking the accuracy of transmission of data in which the received data are returned to the transmitter for comparison with the original data. (Modified from reference [5].)

*Electronics Industries Association Recommended Standard* see *RS-232-C; 422; 423;429*

*Encoder*
A device that reads the position of a robot joint. Absolute encoders output a unique value for every possible joint position. Incremental encoders output values that repeat periodically, and require some additional calibration when the robot is started up. (Modified from reference [26].)

*End-effector*
A device specifically designed for attachment to the mechanical interface to enable the robot to perform its task, [39].

*End-effector identification*
The means of identifying or selecting any end-effector from many end-effectors. Can be done through the establishment of shape, weight, identity code, or other scheme. (Modified from reference [2].)

*End point (in robotics)*
The point at which robotic motion stops along the path of motion, curve, or arc [2].

*End point control*
Any control scheme in which only the motion of the manipulator end point may be controlled and the computer can control the the actuators at the various degrees of freedom to achieve the desired result [5].

*Envelope* see *Work envelope*

*Error control procedures*
Methods for detecting errors and recovering from those that occur in transmitting data. These methods include parity checking, checksums, cyclic-redundancy checks, frame-sequence numbering, and requests for re-transmission. (Modified from reference [2].)

*Error monitoring*
Software and hardware diagnostics for the handling of errors in the control system of a robot. Checks are made of instruction executions, microprocessors, and memory contents [2].

*Error recovery*
Software used to overcome detected error conditions. This may include ways to correct the error conditions, circumvent them, and (in the extreme) to systematically shut the system down until human intervention occurs.

*Expert system*
A computer program, usually based on artificial intelligence techniques, that performs decision functions that are similar to those of a human expert and, on demand, can justify to the user its line of reasoning. Typical applications in the field of robotics are high-level robot programming, planning and control of assembly, and processing and recovery of errors [4].

*Extension*
A linear motion in the direction of travel of the sliding motion mechanism, or an equivalent linear motion produced by two or more angular displacements of a linkage mechanism [5].

*External sensor*
A feedback device that is outside the inherent makeup of a robot system, or a device used to affect the actions of a robot system that is used to source a signal independent of the robot's internal design [5].

*Feedback (in robotics)*
A signal given by an output device (sensor) that is used to drive a control actuator. The part of a closed-loop system that sends information about the state of the phenomena under study or being monitored. The information can include data about a robot's position or speed, forces, temperatures, and the locations of objects that are to be handled by an end-effector. Actual performance can thus be compared with planned performance.

*Feedback control*
The use of feedback to control a robot's movements and the positioning of the end-effector.

*Feedback device*
A device that senses the position of robotic joints and transmits the appropriate data, in either analog or digital form. Such devices include switches, tachometers, encoders, and a host of other sensors. (Modified from reference [2].)

*Feedback system*
A combination of a sensing and a commanding device that can modify the performance of a given act [36].

*Flexible automation*
Refers to the multitask capability of robots; multipurpose, adaptable, reprogrammable.

*Footprint*
The surface area required to mount a robot.

*Fork*
A mounted object with a triangular section removed to aid a robot in removing or dislodging pipette tips, slip-on caps, and other pressure-fit devices.

*Gantry robot* see *Cartesian co-ordinate robot*

*GPIB* see *IEEE-488*

*Gripper*
An end-effector designed for seizing and holding [39].

*Hand* see *End-effector*

*Hardware*
The mechanical, electrical and electronic, pneumatic, or hydraulic devices that compose a computer, controller, robot, workstation, instrument, or peripheral device.

*Heuristic problem solving*
In computer logic, the ability to plan and direct actions to steer toward higher-level goals. This is in contrast to algorithmic problem solving. (Modified from reference [4].)

*Hierarchical control*
A distributed control technique in which the controlling processes are arranged in a hierarchy [5].

*Hierarchy*
A relationship of elements in a structure divided into levels, with those at higher levels having priority or precedence over those at lower levels [5].

*High-level language*
Programming language that generates machine codes from problem or function oriented statements. ALGOL, FORTRAN, PASCAL and BASIC are four commonly used high-level languages. A single functional statement may translate into a series of instructions or subroutines in machine language, in contrast to a low-level (assembly) language in which statements translate on a one-for-one basis [5].

*Hydraulic robot*
Robots that make use of hydraulic servovalves. They are fast, have few moving parts, and can position heavier loads than can pneumatically power robots. Mechanically simple, these robots have an accuracy and reliability associated with electrically actuated robots [2].

*Hysteresis*
The failure of a property that has been changed by an external agent to return to its original value when the cause of the change is removed [30].

*IEEE-488*
The official designation for the General Purpose Instrumentation Bus (GPIB) standard. A bit-parallel, byte-serial bus with well defined control signals. First developed by Hewlett-Packard as HP-IB and later adapted as IEEE-488 by the Institute of Electrical and Electronics Engineers (IEEE). See also *Bus*, Definition 1.

*Instrument*
A device used for observing, measuring, or communicating the state of a quality and which replaces, refines, extends, or supplements human faculties [36].

*Interface (in robotics)*
Those connections of one system that are matched to another system that is distinctly different because of the basic nature of each system. This may be due to the origin of design and construction or due to the basic objectives of each system independently. A shared boundary between system elements defined by common physical or logical interconnection characteristics, signal characteristics, and meanings of interchanged signals. A boundary between the robot and machines, transfer lines, or parts outside of its immediate environment [20].

*I/O*

Abbreviation for: input/output [5].

*ISO*
Abbreviation for: International Organization for Standardization.

*Joint*
A connection between parts or links in a robot that allow motion. The rotational or translational degree of freedom in a robot. The part of a robot's arm that moves. Types of joints include sliding (prismatic) and rotating (revolute).

*Joint co-ordinates*
Robot co-ordinates that specify the position of each joint relative to its arbitrary origin. Prismatic joint co-ordinates are measured in linear quantities such as centimeters or inches; revolute joint co-ordinates are measured in angular quantities such as radians or degrees.

*Joint space*
The space defined by a vector whose components are the angular or translational displacement of each joint in a multi-degree of freedom linkage relative to a reference displacement for each such joint, [5].

*Jointed-arm robot*
A robot whose arm consists of two links connected by 'elbow' and 'shoulder' joints to provide three rotational motions. This robot most closely resembles the movement of the human arm. Also referred to as a revolute robot or anthropomorphic robot. (Modified from reference [20].)

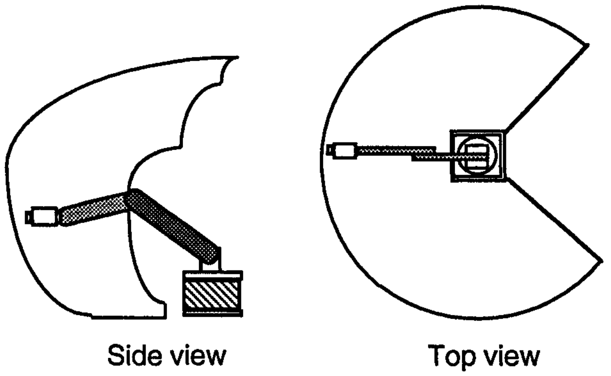

*Figure 5. Jointed-Arm robot and irregular work Envelope.*

*Joystick*
A manually controlled device whose variable position and orientation or applied forces are measured and result in commands to the robot control system [39].

*Kinematics*
The analysis of the geometry of robot motion with respect to a fixed reference coordinate frame as a function of time, without regard to the forces and moments that cause the motion. It studies the relations between the variable joint co-ordinates of the robot mechanism and the positions and orientations of the end-effector. Robot kinematics usually contains two sub-problems: the forward and inverse kinematics. The *forward kinematics* problem is to find the position and orientation of the end-effector with regard to a fixed reference co-ordinate frame, given the joint co-ordinates of the robot mechanism. The *inverse kinematics* problem is to find the appropriate joint co-ordinates for the given position and orientation of the end-effector [3].

*Laboratory unit operation (LUO)*
The building blocks of laboratory-scale operations. Each LUO accomplishes a basic laboratory operation, such as weighing, grinding, conditioning, liquid handling, separating, etc. LUOs are combined in different patterns to process a sample. A particular LUO may use a workstation, such as a liquid handling station or a balance, and the sample may be moved from workstation to workstation.

*LAN* see *Local area network*

*Layer*
In network architecture, a group of services, functions and protocols that is complete from a conceptual point of view, that is one out of a set of hierarchically arranged groups, and that extends across all systems that conform to the network architecture. See figure 6 for an example [35].

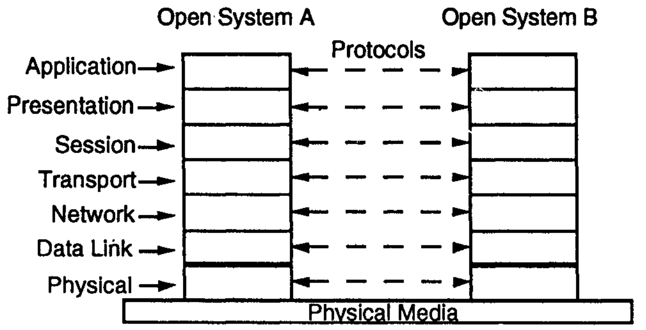

*Figure 6. The seven-layer reference model for open systems interconnection architecture.*

*Limiting load*
The maximum load stated by the manufacturer which can be applied to the mechanical interface without any damage or failure to the robot mechanism under restricted operating conditions [39].

*LIMS*
Laboratory Information Management System: a computerized system designed to provide on-line information about the samples analysed in a laboratory. Information provided may include the current location of each sample in the laboratory, the method and status of each analysis, and experimental data and calculated results (Modified from reference [43].)

*Liquid distribution hand*
An end-effector that is used for pipeting, manifolding, and remote distribution of liquids [1].

*Load*
The force and/or torque at the mechanical interface which can be exerted along the various directions of motion under specified conditions of velocity and acceleration. The load is a function of mass, moment of inertia, and static and dynamic forces supported by the robot [39].

*Load capacity* see *Payload*

*Load deflection*
(1) The difference in position of some point in a body between a non-loaded and an externally loaded condition.
(2) The difference in position of a manipulator end-effector, usually with the arm extended, between a non-loaded condition (other than gravity), and an externally loaded condition. Either or both static and dynamic (inertial) loads may be considered [5].

*Local area network (LAN)*
A communication system that connects a number of computers and their peripherals together to allow information sharing [1].

*Loop*
A programming concept where a sequence of commands is repeatedly executed until some predetermined condition is met.

*Manipulator*
A machine, the mechanism of which usually consists of a series of segments, jointed or sliding, relative to one another, for the purpose of grasping and/or moving objects usually in several degrees of freedom. It may be controlled by an operator, a programmable electronic controller, or any logic system. (Modified from reference [39].)

*Mechanical interface*
The mounting surface at the end of the articulated structure to which the end-effector is attached [39].

*Modular analytical instrument*
An analytical instrument that exhibits the characteristics and behavior of a module. (Modified from references [28] and [45].)

*Module*
An intelligent component that carries out well-defined tasks in a system. It has standardized communications and system interfaces, allowing interchange of data, status information, and material (if appropriate). It may be either a hardware or software entity, but it is designed to be remotely controlled by another machine—not a human. It carries out its operations autonomously, independent of its environment. Simple modules may be combined to form modules of greater complexity or complete systems. (Modified from references [28] and [45].)

*Multichannel pipette hand*
An end-effector that has multiple channels (usually eight) for automatically dispensing liquids, typically used in ELISA (Enzyme Linked Immuno Suppressive Assay) assays.

*Network*
An arrangment of nodes and interconnecting branches [35].

*Network architecture*
The local structure and the operating principles of a computer network [35].

*Normal operating conditions*
The range of environmental conditions (for example temperature, humidity) and other parameters that may influence robot or instrument performance (such as electrical supply instability, electromagnetic fields, etc.) within which the performance of the robot or instrument specified by the manufacturer is valid [39].

*Normal operating state*
The robot state in which the robot is executing its task program as intended [39].

*Off-line programming*
The programming of robotic controllers and computers that involves the writing of task programs, the running of simulations, and the collection and organization of data either away from the robot or when it is not in operation.

*On-line programming*
Robotic programming that makes use of the manipulator. It utilizes the actual robot in order to develop procedures and define the values of data items in a task program. One example of this kind of programming is the record-playback method, which is dependent on an actual robot for testing and demonstration. (Modified from reference [2].)

*Operating system*
The software that controls and aids in the execution of programs within a computer. It handles scheduling, debugging, I/O control, accounting, computations, storage assignments, and data management. The operating system is usually loaded into random-access memory from disk or tape, or stored permanently in read-only memory.

*Operator*
The person designated to start, monitor, and stop the intended productive operation of a robot or robot system. An operator may also interface with a robot for productive purposes [41].

*Optical verification sensor*
A light source/photodetector combination used as an optical sensor to verify an event. (Modified from reference [1].)

*Overshoot*
The degree to which a system response to a step change in reference input goes beyond the desired value [5].

*Path*
An ordered set of poses [39].

*Path control*
The control of point-to-point and continuous-path movements of a robot. This kind of control is programmed and handled automatically. Also called trajectory control [2].

*Payload*
The maximum total mass or weight that can be applied to the end of the robot arm without sacrifice of any of the applicable published specifications of the robot. Also referred to as load capacity [5].

*Pendant*
A hand-held unit linked to the control system with which a robot can be programmed or moved. Also referred to as teach pendant [39].

*Pitch*
The up and down articulation of the robot's wrist. Wrist movement in the vertical plane. The angular rotation of a moving body about an axis, which is perpendicular to its direction of motion and in the same plane as its top side. (Modified from reference [2].)

*Playback accuracy*
(1) The difference between a position command recorded in an automatic control system and that actually produced at a later time when the recorded position is used to execute control.
(2) The difference between actual position response of an automatic control system during a programming or teaching run and that corresponding response in a subsequent run [5].

*Playback robot*
A robot that can repreat a task program which is entered through teach programming [39].

*Pneumatic robot*
A robot containing a pneumatic drive mechanism [1].

*Polar co-ordinate system* see *Spherical co-ordinate system*

*Port*
An inlet or outlet interface connection point of a module used for the interchange of material or information. (Modified from reference [45].)

*Pose*
A combination of position and orientation in space [39].

*Pose-to-pose control*
A control scheme whereby the inputs or commands specify only a limited number of points along a desired path of motion. The control system determines the intervening path segments. A system in which controlled motion is required only to reach a given end point, with no path control during the transition from one end point to the next. Also known as point-to-point control.

*Positioning accuracy* see *Repeatability*

*Power and event control module*
A module that provides programmable control of other laboratory apparatus. Switch closures, input sensors, controlled alternating current (AC) power outlets, analogue/digital converters, and an external direct current (DC) power supply may be provided. (Modified from reference [1].)

*Precision (in robotics)*
Relative to a method of testing, precision is the degree of mutual agreement among individual measurements made under prescribed conditions. *See also Accuracy.* (Modified from reference [20].)

*Product data representation and exchange*
Standard form for the unambiguous representation and exchange of computer-interpretable product information throughout the life of a product, indpendent of any particular computer system. The nature of the description makes it suitable for neutral file exchange, a basis for implementing and sharing product databases, and archiving. The standard is comprised of several parts: description methods, integrated resources, application protocols, abstract test suites, implementation methods, and conformance testing. Also referred to as STEP. [47].

*Programming*
The procedure involved in the generation of robotic algorithms and data, the preparation of programs, and the analysis, problem solving, and logic needed to control a robot. In robotic programming, the programs are dedicated to the control of motors and sensors. The make use of machine language and symbolic code, sometimes shifting between the two for the maintenance of speed and critical operations. (Modified from reference [2].)

*Proximity sensor*
A sensor that determines the presence, position, or distance of an object. Proximity sensors work on the principles of triangulation of reflected light, elapsed time for reflected sound, intensity of induced eddy currents, magnetic fields, back pressure from air jets, or other methods.

*Rack indexing*
The ability to define and access all positions in any rectangular array or rack by teaching a few positions.

*Range*
Maximum distance an arm or wrist can travel; the scope or extent of the travel [2].

*Real-time*
Pertaining to computation or data collection performed in synchronization with the related physical process. (Modified from reference [5].)

*Record-playback robot* see *Playback robot*

*Rectangular co-ordinate system*
Same as Cartesian Co-ordinate system, but applied to points in a plane. *See also Cartesian Co-ordinate system.*

*Rectilinear*
Straight line motion. Moving in sliding motions or along a channel [2].

*Recursive*
The process by which previous steps in a procedure influence those that follow [2].

*Redundancy*
Replication of information or devices in order to improve reliability. (Modified from reference [5].)

*Relative co-ordinate system*
A co-ordinate system whose origin moves relative to fixed co-ordinates [5].

*Relative location*
A location in the robot's work envelope that is relative to some other robot location, frequently an absolute location.

*Reliability*
(1) The probability that a device will function without failure over a specified time period or amount of usage [5].
(2) Reproducibility of required functions. A qualitative combination of accuracy and precision.

*Remote dispensing nozzle*
A movable nozzle that the robot can manipulate to dispense liquids at remote locations. This also refers to a fixed dispensing nozzle to which the robot moves the containers for the addition of liquids.

*Repeatability (in robotics)*
The ability of a robot to reposition itself at a spot to which it is sent or trained to stop. Also called positioning accuracy, it is normally considered a tolerance about a position. Similar in concept to accuracy, it is a different performance characteristic in that it also concerns itself with resolution, the inaccuracies of components, and arbitrary target positions. It is affected by resolution, hysteresis, and inaccuracies in components such as linkages, gears, and beam deflections. As with the capacity of a robot to return to a previously designated position, it describes the positional error of the end-effector when it automatically returns to a previously designated point. It is thus a finer measure of performance than is accuracy. (Modified from reference [2].)

*Resolution*
A function of a robot's control system, resolution specifies the smallest increment of motion by which the system can divide the work envelope. This is either a function of the smallest increment is position that the controller can command or the smallest incremental change in position that the controller can distinguish. Also referred to as spatial resolution [2]).

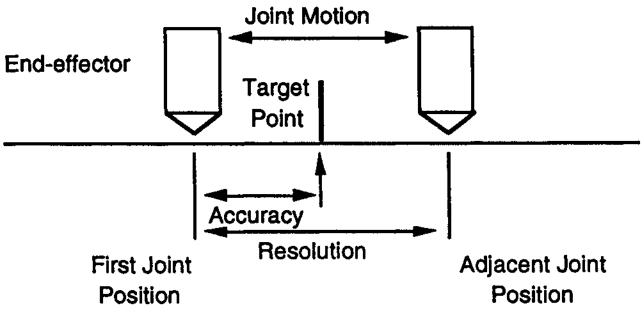

*Figure 7. Resolution.*

*Response time*
The period of time that lapses from the moment an order to start a robotic operation is given and the moment the actual operation begings. This takes into account data transmission and reception, memory access time, and computer processing [2].

*Revolute robot* see *Jointed-arm robot*

*Robot*
An automatically controlled, reprogrammable, multipurpose, manipulative machine with several degrees of freedom, which may be either fixed in place or mobile for use in automation applications [21].

*Robot controller* see *Controller*

*Robot system*
A robot system includes:
(1) The robot (hardware and software) consisting of the manipulator whether mobile or not; power supply and control system.
(2) The end-effector(s).
(3) Any equipment, devices, or sensors required for the robot to perform its task.
(4) Any communication interface that is operating and monitoring the robot, equipment, or sensors, as far as these peripheral devices and supervised by the robot control system [39].

*Robotic classification*
A means of identifying the type of robots. It can be based on physical characteristics, such as hardware construction, degrees of freedom, coordinate systems, and level of sophistication and technology [2].

*Robotics*
The theory and practice of automating tasks being done by humans. This is identified by the interaction of a robot or robotic device and an object. This term comes from the Czech word 'robota', which means work or servant [2].

*Roll*
The circular motion of the robot wrist in a plane perpendicular to the end of the robot arm.

*RS-232-C; 422; 423; 449*
Electronics Industries Association Recommended Standards for interconnection of peripheral devices to computers. RS-232-C, issued in 1969, is the most widely used standard specification for the interface between data terminal equipment and data circuit-terminating equipment that makes use of serial binary data interchange over unbalanced lines. It specifies a maximum range of 12.1 meters, a maximum speed of 20 000 baud. RS-422 specifies the electrical aspects for wideband communications over balanced lines at data rates up to 10 million bits per second. RS-423 calls for the same for unbalanced lines at rates up to 100 000 bits per second. RS-449 defines the mechanical specifications for connectors and the functions of each circuit. It takes advantage of recent advances in integrated circuit design, reduces cross talk between between interchange circuits, permits greater distances between equipment, and allows even higher data signaling rates: 2 million bits per second. In specifying the functional and mechanical aspects of interfaces, it allows connectors that have 37 pins or 9 pins, instead of a single 25-pin connector commonly used with RS-232-C. (Modified from reference [2].)

*Sensor*
A device that measures some property (for example, receives a stimulus) of the real world and informs (for example, responds to) its system about the result of the measurement. The lack of a sensor implies a lack of input to the system about the property in question. The sensor is a subsystem for gaining information about the real world. A sensor includes the transducer and the transmitter as part of its design [23].

*Sensory control*
A control scheme whereby the robot motion or force is adjusted in accordance with outputs of external sensors [39].

*Serial processing*
Processing several samples through a procedure simultaneously. At any given moment each sample will be in a different stage of the procedure. Serial processing allows uniform sample history, maximizes hardware utilization, and minimizes hardware capacity requirements. In contrast to *batch processing*.

*Servocontrolled robot*
A robot driven by servomechanisms. Such a robot is capable of stopping at or moving through a practically unlimited number of points in executing a programmed trajectory. (Modified from reference [5].)

*Servomechanism*
An automatic control mechanism consisting of a device driven by a signal that is a function of the difference between commanded position and/or rate, and measured actual position and/or rate. (Modified from reference [5].)

*Shoulder*
The joint, or pair of joints, that connect the arm to the base [5].

*Slide*
A type of articulation in a robot; a translational degree of freedom [2].

*Software*
(1) Intellectual creation comprising the programs, procedures, rules, and any associated documentation pertaining to the operation of a data processing system [31].
(2) The programs and attendant information needed needed to run a computer or robot; programs are run on a computer or by a controller. The software can be supplied in full or be open-ended, i.e., the user can write his own sequences and applications programs. (Modified from reference [2].)

*Solenoid*
An electromagnet with a movable core, or plunger, that, when energized, can move a small mechanical part a short distance. Often used to activate valves. (Modified from reference [5].)

*Spatial resolution* see *Resolution*

*Speed see Velocity*

*Spherical co-ordinate robot*
A robot, the manipulator degrees of freedom of which are defined primarily by spherical coordinates. (Modified from reference (5).)

*Spherical coordinate system*
A coordinate system where two dimensions are angles, and the third is the linear distance from the point of origin. These three coordinates specify a point on a sphere. Also referred to as a polar co-ordinate system. (Modified from reference [5].)

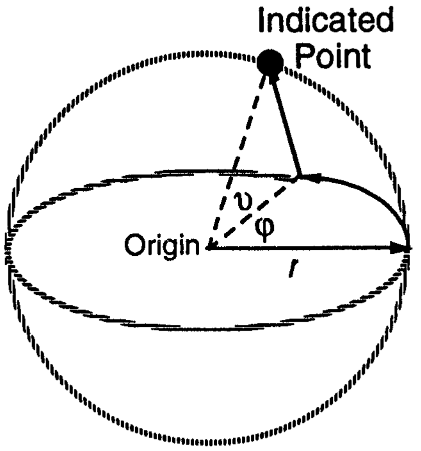

*Figure 8. Spherical co-ordinate system.*

*Stability*
A major and critical property of a robot. The stable nature at the end-effector. The lack of oscillation or undesired motion about any robotic axis.

*Static*
Refers to a state in which a quantity does not change appreciably within an arbitrarily long time interval [5].

*Static compliance*
The maximum amount of displacement per unit of load applied to the mechanical interface [39].

*STEP* see *Product data representation and exchange*

*Stop-point*
Command pose (taught or programmed) which shall be attained by the axes of the robot with a velocity command equal to zero and no deviation in positioning [39].

*Subroutine*
A computer program portion which performs a secondary or repeated function, such as printing or sorting. A subroutine is executed repeatedly as required by the main program [5].

*Syringe hand*
An end-effector that has a motor-driven syringe attached for the transfer of liquids. Often used in conjunction with pipeting operations. (Modified from reference [1].)

*System*
Equipment that as a group forms a whole. A group of devices that form a network for a common purpose or for a common distribution method. Four attributes are associated with a system: the number of units that make up the whole, the relationship among the units, the objective or goal of the system, and its adaptability to the environment. (Modified from reference [2].)

*Systems engineering*
The technique of optimizing the design, installation, and execution of large-scale systems. Makes use of scientific laws and empirical rules [2].

*Tactile sensor*
A sensor that is sensitive to touch. Associated usually with the end-effector of the robot which senses contact with an object. Classified into touch and stress types, this sensor may be a microswitch, strain gage, or other conductive device. (Modified from reference [2].)

*Task program*
The set of instructions for motion and auxiliary functions that define the specific intended task of the robot system. This type of program is normally generated by the user [39].

*Teach*
To move a robot to or through a series of points that are stored in the robot controller for the robot to perform its intended task. (Modified from reference [5].)

*Teach pendant* see *Pendant*

*Teach programming*
Programming performed by:
(1) Manually leading the robot end-effector.
(2) Manually leading a mechanical simulating device.
(3) Using a pendant to move the robot through the desired actions [39].

*Terminal*
(1) Any fitting attached to a circuit or device for convenience in making electrical connections.
(2) An interface device containing a cathode ray tube and a keyboard, for communicating with a computer or robot controller [5].

*Time out*
An event that occurs at the end of a predetermined period of time that began at the occurrence of another specified event [33].

*Tolerance*
A specified allowance for error from a desired or measured quantity [5].

*Top-down design*
The development of software for robots in stages or increments, from the highest level to the lowest, and from the general to the particular. Its aim is the creation of logical design that can then be implemented by structured programming. Top-down design is a formal mechanism for breaking complex process designs into functional descriptions, for reviewing progress, and for allowing modifications [2].

*Trajectory*
A path in time [39].

*Trajectory control* see *Path control*

*Transducer*
A transducer converts input energy in one form to output energy of another. The transducer is a component of a sensor (23).

*Velocity*
Distance traveled in a specified amount of time: $v = \mathrm{d}r/\mathrm{d}t$. The rate at which the end of the robot arm reaches its positions. The rate at which a gripper grasps an object. The linear and angular rate at which a joint moves, related to the torque and mass. (Modified from reference [2].)

*Work envelope*
The set of points that represents the maximum extent and reach of a robot's wrist. It excludes the end-effector because manufacturers cannot predict the shape or size of end-effector eventually used by the robot. The envelope can be rectangular, cylindrical, spherical, or irregular. Shapes are determined by the length of the robot's links and the arrangement of the joints. Any envelope has three parameters associated with it: the horizontal arm sweep or the degrees of rotation about the center, the vertical motion of the arm, and the radial extension of the arm as measured from the center axis. (Modified from reference [2].)

*Workstation*
An apparatus that performs some automated function or with which a laboratory unit operation is performed.

*Wrist*
The set of rotary joints to which a robot's end-effector is attached. It may exhibit compliance (can be used with many different end-effectors), overload protection, and strength. (Modified from reference [2].)

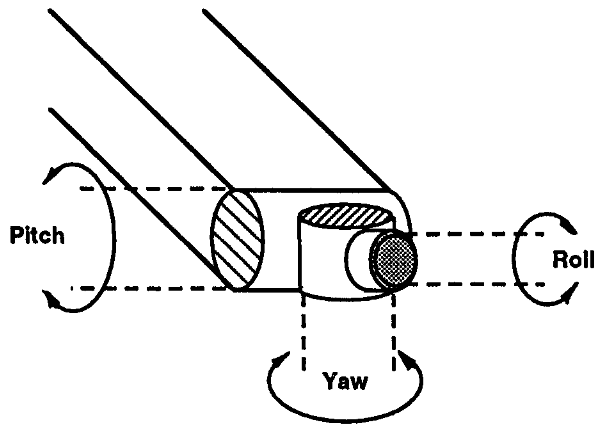

*Figure 9. Typical wrist articulation.*

*Yaw*
The side-to-side rotary motion of the robot wrist which is perpendicular to the line of motion and the top side of the wrist. (Modified from reference [5].)

### Acknowledgment

The authors thank the following individuals who provided detailed review and expertise in the preparation of this glossary:

Dr R. E. Bareiss, Hüthig & Wepf Verlag; Dr Tony Beugelsdijk, Los Alamos National Laboratory; Dr Colin L. Graham, University of Birmingham; John Helfrich, Zymark Corporation; Dr William Horwitz, IUPAC; Dr Jeffrey Hurst, Hershey Foods; Dr Thomas L. Isenhour, Duquesne University; Dr Jack Jamerson, Union Carbide Corporation; Dr Gary W. Kramer, National Institute of Standards and Technology; Dr Rich Lysakowski, Digital Equipment Corp., Dr A. D. McNaught, Royal Society of Chemistry; Fred Proctor, National Institute of Standards and Technology; J. Christopher Rigg; Dr Frank Settle, Virginia Military Institute; Anders J. Thor, SIS-Standardiseringskommissionen i Sverige; Dr R. L. Tranter, Glaxo Manufacturing Services; Dr Fritz Weber, F. Hoffman-LaRoche Ltd; Dr Ted Weglarz, Dow Chemical USA; Dr Gerhard Wieland, MERCK; and Jim Zdunek, Kraft-General Foods Research and Development.

### References

1. Hurst, Jeffrey W. and Mortimer, James W. *Laboratory Robotics: A Guide to Planning, Programming, and Applications* (VCH Publishers, New York, 1987).
2. Waldman, Harry, *Dictionary of Robotics* (Macmillan Pub. Co., New York, 1985).
3. Dorf, Richard C., *International Encyclopedia of Robotics* (Wiley Interscience, New York, 1988).
4. Nof, Shimon Y., *Handbook of Industrial Robotics* (John Wiley, New York, 1985).
5. *RIA Robotics Glossary* (Robot Institute of America, Dearborn, MI, 1984).
6. Miller, Rex, *Fundamentals of Industrial Robots and Robotics* (PWS-KENT Pub. Co., Boston, 1988).
7. Hunt, V. Daniel, *Smart Robots: A Handbook of Intelligent Robotic Systems* (Chapman and Hall, New York, 1985).
8. Scott, Peter (ed.), *The World Yearbook of Robotics Research and Development* (Gale Research Co., Detroit, MI, 1986).
9. Little, James N., *Trends in Analytical Chemistry*, **2** (1983) 103–105.
10. Dessy, Raymond, *Analytical Chemistry*, **55** (1983), 1100A–1114A.
11. Dessy, Raymond, Owens, Grover, Eckstein, Rodney, Franz, Tom, Dittenhafer, Mark and McLean, James, *Analytical Chemistry*, **55**, 1233A–1242A, 1983.
12. Lento, Harry, *Robotics Today*, **6** (1984), 43–47, 1984.
13. Boman, Stuart A., *Analytical Chemistry*, **56** (1984), 265A-265A-271A.
14. Isenhour, Thomas, *Journal of Chemical Information and Computer Sciences*, **25** (1985), 292–295, 1985.
15. Binkley, David P., *American Laboratory* (February, 1986), 68–74.
16. Brown, Richard K., *Chemistry in Britain* (July 1986), 640–642.
17. Schmidt, Gary J. and Dong, Michael W. *American Laboratory* (February 1987), 62–72.
18. Pruskowski, E. D., Yates, A. and Salit, M. L., *American Laboratory* (March 1987), 90–96.
19. Groover, Mikell P., Weiss, Mitchell, Nagel Roger N. and Odrey, Nicholas G., *Industrial Robotics: Technology, Programming, and Applications* (McGraw-Hill, New York, 1986).
20. Hunt, V. Daniel, *Robotics Sourcebook* (Elsevier, New York, 1988).
21. *Manipulating Industrial Robots: Vocabulary* (International Organization for Standardization, ISO/TR 8373:1988).
22. Short, Kenneth L., *Microprocessors and Programmed Logic* (Prentice-Hall, Englewood Cliffs, NJ, 1981).
23. Helmers, Carl, *Sensors*, **3** (1986), 3.
24. Hamacher, V. Carl, Vranesik, Zvonko G. and Zaky, Safwat G., *Computer Organization* (McGraw-Hill, New York, 1978).
25. Craig, John J., *Introduction to Robotics* (Addison-Wesley, Reading, MA, 1986).
26. Kafrissen, Edward, and Stephens, Mark, *Industrial Robots and Robotics* (Reston Pub. Co., Reston, VA, 1984).
27. *Laboratory Robotics Handbook* (Zymark Corp., Hopkinton, MA, 1988).
28. Kingston, H. M. (Skip), *Analytical Chemistry*, **61** (1989) 1381.
29. Guilbault, G. C. and Hjelm, M., *Nomenclature for Automated and Mechanized Analysis: Recommendations 1989* (International Union of Pure and Applied Chemistry, 1989).
30. *American Heritage Dictionary of the English Language* (Houghton Mifflin Co., Boston, MA, 1970).
31. *Data Processing—Vocabulary. Part 01: Fundamental Terms* (International Organization for Standardization, ISO 2382/1:1984).
32. *Data Processing—Vocabulary. Section 05: Representation of Data* (International Organization for Standardization, ISO 2382/V:1974).
33. *Data Processing—Vocabulary. Part 09: Data Communication* (International Organization for Standardization, ISO 2382/9:1984).
34. *Information Processing Systems—Vocabulary. Part 11: Processing Units* (International Organization for Standardization, ISO 2382-11: 1987).
35. *Information Processing Systems—Vocabulary. Part 18: Distributed Data Processing* (International Organization for Standardization, ISO 2382-18: 1987).
36. Gold, Victor, Loening, Kurt L., McNaught, Alan D. and Sehmi, Pamil, *Compendium of Chemical Terminology: IUPAC Recommendations* (Blackwell Scientific Publications, Oxford, 1987).
37. Horwitz, W., *Pure and Applied Chemistry*, **62** (1990), 1193–1208.
38. Guilgault, G. G. and Hjelm, M., *Pure and Applied Chemistry*, **61** (1989), 1657–1664.
39. *Manipulating Industrial Robots: Vocabulary* (International Organization for Standardization, ISO DIS 8373, 1993).
40. *American National Standard for Industrial Robots and Robot Systems: Safety Requirements* (American National Standards Institute, ANSI/RIA R15.06-1992).
41. *RIA Glossary of Robot Terms* (Robotics Industries Association, Draft revision of 1984 edn).

42. *Information Processing Systems—Vocabulary. Part 04: Organization of Data* (International Organization for Standardization, ISO 2382-4: 1987).
43. GIBBON, GERST A., *TrAC, Trends Analytical Chemistry*, **3**, 36–38, 1984.
44. BEUGELSDIJK, T. J., ERKKILA, T. H., ELLING, J. W., and HOLLEN, R. M., *Environmental Test Analysis*, **68** (1993).
45. SALIT, M. L., GUENTHER, F. R., KRAMER, G. W. and GRIESMEYER, M. J., *Analytical Chemistry* (in press).
46. KRAMER, G. W., *American Laboratory*, **10** (1993).
47. *Product Data Representation and Exchange. Part 1: Overview and Fundamental Principles* (International Organization for Standardization, ISO DIS 10303-1, 1993).

*Appendix. Reviewers of recommendations for nomenclature in laboratory robotics and automation.*

Dr R. E. Bareiss
Die Makromolekulare Chemie
Hüthig & Wepf Verlag
Hegelstrasse 45
D-6500 Mainz
Germany

Dr Tony Beugelsdijk
Los Alamos National Laboratory
PO Box 1663, MS E537
Los Alamos, NM 87545

Joe Cross
Phillips Petroleum
140 PL
Bartlesville, OK 74004

Dr Jim Curley
Pfizer, Inc.
Eastern Point Rd.
Groton, CT 06340

Dr Colin L. Graham
School of Chemistry
University of Birmingham
Edgbaston
Birmingham B15 2TT
United Kingdom
Secretary: Analytical Chemistry Division, IUPAC

John Helfrich
Zymark Corporation
Zymark Center
Hopkinton, MA 01748

Dr Jeffrey Hurst
Hershey Foods, P.O. Box 575
Hershey, PA 17033
Editor: *Laboratory Robotics and Automation*, VCH Publishers

Dr Thomas L. Isenhour
Department of Chemistry
Duquesne University
Pittsburgh, PA 15282
Editor: *Journal of Chemical Information and Computer Sciences*, American Chemical Society (at time of review)

Dr Jack Jamerson
Union Carbide Corporation
Inorganic Analysis Skill Center
PO Box 8361
South Charleston, WV 25303

Dr Gary W. Kramer
National Institute of Standards & Technology
Center for Analytical Chemistry
Consortium on Automated Analytical Laboratory Systems
Gaithersburg, MD 20899

Dr Rich Lysakowski
Digital Equipment Corp.
Four Results Way, MRO4-2/C17
Marlboro, MA 01752

Dr R. D. McNaught
Royal Society of Chemistry
Thomas Graham House
Science Park, Milton Road
Cambridge CB4 4WF
United Kingdom
Secretary: Interdivisional Comm. on Nomenclature and Symbols (IDCNS), IUPAC

Fred Proctor
National Institute of Standards & Technology
Center for Manufacturing Engineering
Robot Systems Division
Gaithersburg, MD 20899

J. Christopher Rigg
Langhoven 57
6721 SL Bennekom
Netherlands

Dr Grant Schoenhard
G. D. Searle Corporation
4901 Searle Parkway
Skokie, IL 60077

Dr Frank Settle
Department of Chemistry
Virginia Military Institute
Lexington, VA 24450
Column Editor: *Journal of Chemical Education*, American Chemical Society

Dr William Sonnefeld
Senior Chemist
Eastman Kodak Company
B-49, 1st Floor, Kodak Park
Rochester, NY 14562

Anders J. Thor
SIS-Standardiseringskommissionen i Sverige
Box 3295
S-103 66 Stockholm
Sweden
Secretariat: ISO/TC 12

Dr R. L. Tranter
Glaxo Manufacturing Services
Harmire Road, Barnard Castle
County Durham DL12 8DT
United Kingdom

Dr Fritz Weber
F. Hoffman-LaRoche Ltd
Dept. of Vitamin and Nutrition Research
Building 73/3C
Grenzacherstr. 124
CH-4002 Basle
Switzerland

Dr Ted Weglarz
Dow Chemical USA
Michigan Division
Building 574
Midland, MI 48667

Dr W. Wegscheider
Institut für Analytische Chemie, Mikro-und Radiochemie.
Technische Universität Graz
Technikerstrasse 4, A-8010
Graz, Austria
Editor: *Chemometrics and Intelligent Laboratory Systems*,
American Chemical Society

Dr Gerhard Wieland
MERCK
Frankfurter Strasse 250
Postfach 41 19
6100 Darmstadt 1
Germany

Jim Zdunek
Kraft-General Foods Research & Development
Kraft Technical Center
801 Waukegan Road
Glenview, IL 60025

